# Incidence, risk factors, and prognosis of acute kidney injury in mechanically ventilated dogs and cats

**DOI:** 10.3389/fvets.2026.1788257

**Published:** 2026-05-01

**Authors:** Zhi H. Hsu, Alex M. Lynch, Ronald H. Li, Bernie Hansen, Yu Ueda

**Affiliations:** Department of Clinical Sciences, College of Veterinary Medicine, North Carolina State University, Raleigh, NC, United States

**Keywords:** azotemia, diuretics, positive pressure ventilation, renal failure, respiratory failure

## Abstract

**Objective:**

To evaluate the incidence, risk factors, and prognostic impact of acute kidney injury (AKI) in dogs and cats managed with mechanical ventilation (MV).

**Design:**

Retrospective single-institution study (Jan 2015–Aug 2023).

**Setting:**

University teaching hospital.

**Animals:**

96 animals, including 81 dogs and 15 cats, undergoing MV management.

**Measurements and main results:**

The incidence of AKI in this animal cohort was 26% (25/96). There was no significant difference in AKI incidence between dogs and cats (*p* > 0.99). The mortality rate of animals with AKI was 64% (16/25) while those without AKI was 50.7% (36/71) (*p* = 0.35). AKI development was not significantly associated with the survival to discharge rate, while SpO_2_ measured before (*p* = 0.01) and after initiation of MV (*p* = 0.0079) and duration of hospitalization (*p* < 0.0001) were significantly associated with the survival to discharge rate. In multivariable logistic regression analysis, age, baseline serum creatinine, congestive heart failure status, and duration of MV and hospitalization were not independently associated with AKI development.

**Conclusion:**

AKI was common in animals managed with MV. However, the development of AKI was not significantly associated with the survival to discharge rate in this cohort.

## Introduction

Mechanical ventilation (MV) is a life-saving supportive therapy, and its clinical application is widely accepted in veterinary medicine (1–5). Hypoxemia, hypoventilation, and respiratory muscle fatigue, secondary to failure of the respiratory, cardiovascular, and neurological systems are common indications of MV, especially when animals do not respond to the conventional therapy (2, 4, 6).

Complications related to MV previously reported in companion animals include hypothermia, hypotension, cardiac arrhythmias, fluid overload, pneumonia, oliguria, and acute kidney injury (2, 3). These complications may be associated with the underlying disease processes, level of respiratory support required, or a combination of these factors (7–15). In dogs and cats managed with MV for left-sided congestive heart failure (CHF), the development of azotemia or oliguria has been reported to be negatively associated with survival (6, 16).

Acute kidney injury is defined as an acute insult to the renal parenchyma, reflected by the accumulation of uremic toxins with or without decreased urine production (17). Clinically, AKI is diagnosed by documentation of an acute decrease in kidney function, within a 48-h period, or an increase in kidney injury markers, such as urinary cystatin B (17–19). In human patients, AKI development during MV is frequently associated with baseline illness severity and level of respiratory support required, as well as ICU-acquired complications such as sepsis and organ dysfunction (13, 20, 21). As a result, a meta-analysis revealed a threefold increase in the odds of developing AKI in patients who required MV compared with those who did not (14).

In veterinary medicine, a recent study (20) reported that approximately 6% of dogs and cats managed with MV developed AKI, which is substantially lower than the reported incidence of 20–30% in people (8, 13, 21, 22). A critical knowledge gap exists regarding the true incidence of AKI in dogs and cats undergoing MV. Additionally, improved characterization of the prognosis and risk factors associated with AKI in animals undergoing MV, as early recognition and prevention may improve clinical outcomes in these patients. Therefore, the primary objective of this study was to determine the incidence and identify possible risk factors for AKI in dogs and cats undergoing MV. We also sought to evaluate whether AKI development during MV would be associated with clinical outcomes, including the rates of MV weaning and hospital discharge. We hypothesized that AKI would occur more frequently during MV compared to previously reported in dogs and cats, and that its development would be associated with increased mortality in mechanically ventilated animals.

## Materials and methods

Dogs and cats treated with MV between January 2015 and August 2023 at the North Carolina State University Randall B. Terry, Jr. Companion Animal Veterinary Medical Center were screened for inclusion in this study. Enrolled animals were identified by searching the electronic medical record system using the “ventilator set-up” billing code. A total of 149 animals, including 123 dogs and 26 cats, were identified. The following data were collected from medical records for each patient: age, sex, breed, primary clinical indications for initiating MV, MV duration, length of hospitalization, and serum creatinine concentrations s[Cr]. Animals with incomplete data were excluded from further analysis.

Animals with at least two s[Cr] measurements within 48 h were included in this study. s[Cr] was measured within 24 h prior to initiating MV. The second and third measurements were then obtained within 48 h after the initial measurement either during MV or after the animals had been weaned off from MV. Acute kidney injury was defined as an increase in s[Cr] of ≥ 0.3 mg/dL within 48 h, following the International Renal Interest Society (IRIS) AKI grading system (17). Animals without at least two s[Cr] within a 48-h period during MV were excluded from AKI-related analyses. Animals with an elevated s[Cr] due to pre-existing kidney disease prior to MV management were not excluded from this study, but they were diagnosed with AKI only when s[Cr] was increased by more than 0.3 mg/dL from the baseline value within 48 h. When animals had three creatinine measurements and s[Cr] was elevated at the second time point (within 24 h), they were not diagnosed with AKI if s[Cr] subsequently decreased to within 0.3 mg/dL of the baseline value. Animals with lower urinary tract obstruction were excluded from further analysis. Urinalysis data, including urine specific gravity (USG), were recorded when available.

During the study period, MV management was initiated, maintained, and discontinued by American College of Veterinary Emergency and Critical Care Diplomates or by residents under their direct supervision. Personnel involved in the care of mechanically ventilated patients were trained under a standardized protocol at the author’s institution, which is based on standardized treatment recommendations in the literature (23–25). Peripheral oxygen saturation (SpO_2_) was measured within 6 h before initiating MV management (pre-MV SpO_2_), and within 24 h after the MV initiation (MV SpO_2_) was recorded. If multiple SpO2 values were available, the value measured immediately prior to the initiation of MV was used for analysis. When multiple SpO2 values were available after MV initiation, the first stable values recorded along with the corresponding FiO2 was used for the analyses. The SpO2-to-FiO2 ratio (SF ratio) was calculated as the peripheral oxygen saturation (SpO2) divided by the fraction of inspired oxygen (FiO2), both prior to (pre-MV SF ratio) and after initiation of MV (MV SF ratio), when both values were available. Positive end-expiratory pressure (PEEP) and peak inspiratory pressure (PIP) were set and recorded, and the average over the first 6 h of MV was used for analysis in the present study. Blood pressures were measured either directly or indirectly. In addition, the use of vasopressors and inotropes during MV was recorded when data were available. Vasopressor was administered to hypotensive patients without evidence of hypovolemia. Ionotrope was considered with ultrasonographic evidence.

Outcome variables included development of AKI, successful withdrawal of MV, and survival to discharge. The duration of MV and length of hospitalization were determined based on entries in the patient care billing system and the treatment records. Hospitalization length was calculated based on the hospitalization charge and the treatment records.

### Statistical analysis

Continuous variables were assessed for normality using visual inspection of histograms and Shapiro–Wilk test. Nonparametric data were reported as median and interquartile range (IQR). Parametric data were reported as mean and standard deviation (SD). Categorical variables were reported as counts and percentages. Comparisons between AKI groups for continuous variables, including age, MV duration, length of hospitalization, baseline s[Cr], pre-MV SpO_2_, MV SpO_2_, pre-MV SF ratio, MV SF ratio, USG were performed using the student’s t-test or Mann–Whitney U test. Associations between categorical variables, including sex, AKI status, indications of MV, CHF status, weaning from MV rate, and survival outcome, were evaluated using the chi-square test or Fisher’s exact test. Subgroup analyses were performed separately for dogs and cats, for animals with and without CHF, and for animals with normal and elevated baseline s[Cr] to evaluate potential species- or disease-specific associations with AKI development and survival outcome.

Univariable logistic regression analyses were performed to determine factors associated with AKI development and survival to hospital discharge. Predictor variables included age, sex, CHF status, baseline s[Cr], MV duration, length of hospitalization, pre-MV SpO_2_, MV SpO_2_, pre-MV SF ratio, MV SF ratio, PEEP, PIP, MAP, and the use of vasopressors. AKI status was also added as a predictor variable for survival. Odds ratios (ORs) with corresponding 95% confidence intervals (CIs) were calculated. Multivariable modeling was also performed by including the independent variables with *p*-values < 0.2 in the univariable logistic regression analysis. All statistical analyses were performed using commercially available statistical software (Prism 10.6.1, GraphPad, Boston). A two-sided *p* value < 0.05 was considered statistically significant.

## Results

### Study population

A total of 149 animals managed with MV were identified, consisting of 123 dogs and 26 cats. Overall survival to hospital discharge was 31.5% (47/149), while 68.5% (102/149) of animals died or were euthanized during hospitalization. Of these 149 animals, 53 cases were excluded due to incomplete medical records or the absence of repeated s[Cr] measurements within 48 h during MV management. As a result, a total of 96 cases (81 dogs and 15 cats) were included for the analyses.

The median subject age was 8 years (IQR: 4–10). The cohort consisted of 48 males (12 intact, 36 neutered) and 48 females (2 intact and 46 spayed). The most common indications for initiating MV were primary hypoxemia (67.7%; 65/96), followed by hypoventilation (17.7% 17/96), mixed hypoxemia and hypoventilation (5.2%; 5/96), and other causes (9.4%; 9/96). In animals with primary hypoxemia, the most common underlying disease diagnosed was CHF (21.9%; 21/96 cases), followed by aspiration pneumonia (14.6%; 14/96) (Supplementary Table 1). The median duration of MV management was 35 h (IQR: 17–61), and the median length of hospitalization was 4 days (IQR: 3–5). The overall MV weaning rate was 50% (48/96), and the discharge from hospital rate was 45.8% (44/96), while 54.2% (52/96) died or were euthanized during hospitalization.

### Incidence and severity of AKI

**Figure 1:**
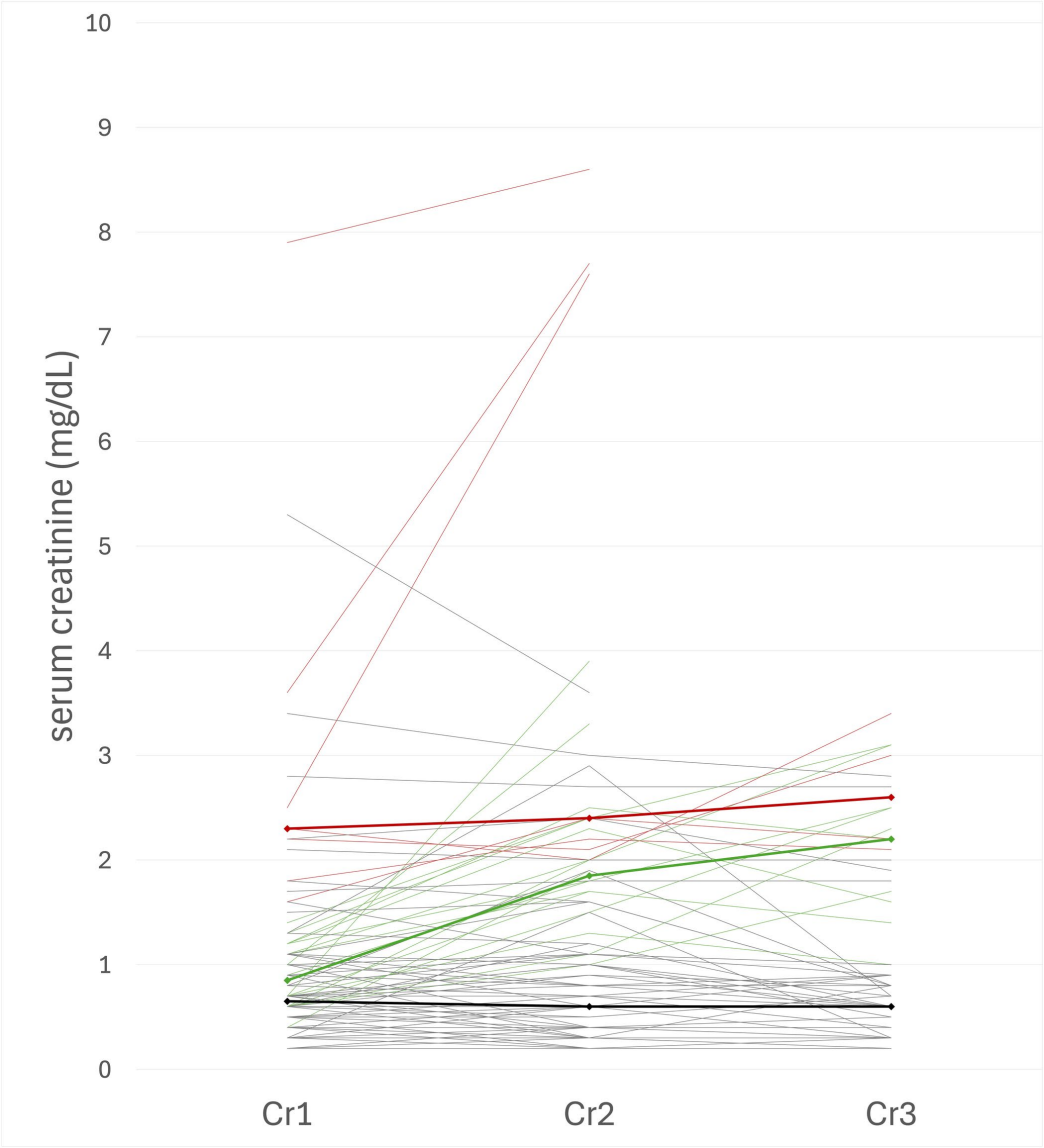
Serum creatinine [s(Cr), mg/dL] values plotted at three time points (Cr1–Cr3) in all animals (*n* = 96) with acute kidney injury with elevated baseline s[Cr] (AKI) (red), AKI with normal baseline s[Cr] (green), and without AKI (blue). Serum creatinine concentration was measured at the first time point (Cr1) within 24 h before initiating mechanical ventilation (MV). The second (Cr2) and third time points (Cr3) were measured within 48 h after the first time point (Cr1). Each line represents one animal. Lines terminate where measurements were unavailable. Bold lines represent the median s[Cr] trend for each group: AKI with elevated baseline (red), AKI with normal baseline (green), and without AKI (black).

Acute kidney injury (AKI) was identified in 25 of 96 animals (26%) (Figure 1). In dogs, AKI was identified in 21 out of 81 cases (25.9%). In cats, AKI was identified in 4 out of 15 cases (26.7%) (Table 1). There was no significant difference in the incidence of AKI between dogs and cats (*p* > 0.99). Based on the IRIS AKI grading system, 8% (2/25) of animals with AKI were classified as grade I, 56% (14/25) as grade II, 24% (6/25) as grade III, 12% (3/25) as grade IV, and no animals were classified as grade V.

**Table 1:** Baseline characteristics of animals (*n* = 96) managed with mechanical ventilation, categorized by the development of acute kidney injury.

Baseline characteristics (all animals)		No AKI		AKI	
Dogs	% (n/n)	74.1	(60/81)	25.9	(21/81)
Cats	% (n/n)	73.3	(11/15)	26.7	(4/15)
Overall	% (n/n)	74	(71/96)	26	(25/96)
Age	Years	8	(4–10)	9	(5.6–10.6)
Sex Male/Female	n/n	32/39		16/9	
Baseline s[Cr]	mg/dL	0.65^a^	(0.4 - 1.0)	1.1^a^	(0.7–1.7)
Pre-MV SpO2%	%	94.5	(91.8–97.3)	96	(93.3–100)
MV SpO2	%	98	(96–100)	100	(94–100)
Pre-MV SF ratio	-	242.5	(230–457.1)	241.2	(230.6–300.9)
MV SF ratio	-	100	(96–108.8)	100	(98.5–112.5)
PEEP	mmHg	5.5	(4–8)	6	(5–7)
PIP	mmHg	17	(13.9–21)	16.5	(15–21.8)
MAP	mmHg	84	(78–102)	85	(72–103.5)
Vasopressor use	% (n/n)	38.2	(13/34)	61.5	(8/13)
Duration of MV hours	Hours	40	(16.3–65.8)	27	(20–37)
Length of hospitalization days	Days	4	(3–6)	4	(2–5)

Continuous variables were reported as median (interquartile range) or mean (standard deviation). Categorical variables were reported as percentage (*n*/total number).

AKI, acute kidney injury; s[Cr], serum creatinine concentration; Pre-MV SpO_2_, SpO_2_ measured prior to initiating mechanical ventilation management, MV SpO_2_, SpO_2_ measured within 24 h after the initiation of mechanical ventilation management; MV, mechanical ventilation; PEEP, positive end-expiratory pressure; PIP, peak inspiratory pressure; MAP, mean arterial pressure.

^a^*P* = 0.0003.

Of these 96 animals, 80 animals had normal baseline s[Cr], and 16 animals had pre-existing azotemia with baseline s[Cr] ≥ 1.6 mg/dL. Of the animals with normal baseline s[Cr], 22.5% (18/80) of animals developed AKI, while 43.75% (7/16) of animals with elevated baseline s[Cr] developed AKI with further elevation of s[Cr] ≥ 0.3 mg/dL within 48 h after initiation of MV management (Table 2). The baseline s[Cr] was significantly higher in animals with AKI than in animals without AKI (*p* = 0.0003).

**Table 2:** Baseline characteristics of animals (*n* = 80) with normal baseline s[Cr] before initiation of mechanical ventilation, categorized by AKI development.

Baseline characteristics (normal baseline sCr)		No AKI		AKI	
Dogs	% (n/n)	77.1	(54/70)	22.9	(16/70)
Cats	% (n/n)	80	(8/10)	20	(2/10)
Overall	% (n/n)	77.5	(62/80)	22.5	(18/80)
Age	Years	7.6	(3.8–10)	8	(6–10)
Sex male/female	n/n	25/37		6/12	
Baseline s[Cr]	mg/dL	0.6^a^	(0.4 – 0.8)	0.85^a^	(0.6–1.2)
CHF	% (n/n)	9.7	(6/62)	27.8	(5/18)
Pre-MV SpO2	%	95	(92–97.5)	96.5	(90.5–100)
MV SpO2	%	98	(96–100)	100	(94.5–100)
Pre-MV SF ratio	-	242.5	(235–466.7)	242.5	(235.6–412.5)
MV SF ratio	-	100	(96.5–166.7)	100	(97–108.8)
PEEP	mmHg	5.5	(4–8)	6	(3.5–8.5)
PIP	mmHg	17	(13.8–21)	20.5	(15.5–24.3)
MAP	mmHg	84	(78–103)	87	(76–115.5)
Vasopressor use	% (n/n)	34.5	(10/29)	62.5	(5/8)
Duration of MV	Hours	40	(16–66)	24	(20–36)
Length of hospitalization	Days	4	(3–6)	4	(2–5)

Continuous variables were reported as median (interquartile range) or mean (standard deviation). Categorical variables were reported as percentage (*n*/total number).

AKI, acute kidney injury; s[Cr], serum creatinine concentration; Pre-MV SpO_2_, SpO_2_ measured prior to initiating mechanical ventilation management, MV SpO_2_, SpO_2_ measured within 24 h after the initiation of mechanical ventilation management; MV, mechanical ventilation; PEEP, positive end-expiratory pressure; PIP, peak inspiratory pressure; MAP, mean arterial pressure.

^a^*P* = 0.0018.

Urinalysis data were available in 16 of 96 animals. The median USG was 1.019 (IQR: 1.015–1.036). USG was not significantly different between the AKI group (1.020, IQR: 1.018–1.024) and the Non-AKI group (1.018, IQR: 1.013–1.037) (*p* = 0.69). Urine sediment examination results were available in 15 animals, with abnormalities identified in 10 cases (66.7%), most commonly hematuria (40%; 6/15 cases) and pyuria (33.3%; 5/15 cases).

### Risk factors for AKI development

The development of AKI during MV was not significantly different among animals with different indications of MV (*p* = 0.14). However, the development of AKI during MV was significantly associated with concurrent CHF (OR 3.64, 95% CI 1.36–9.68, *p* = 0.022). In animals with CHF, 47.6% (10/21) of animals developed AKI, while 20% (15/75) of animals without CHF developed AKI. There was no significant difference in age (*p* = 0.14), sex (*p* = 0.16), duration of MV (*p* = 0.22), and length of hospitalization (*p* = 0.2) between animals with and without AKI. The pre-MV SpO_2_ (*p* = 0.37), MV SpO_2_ (*p* = 0.99), pre-MV SF ratio, MV SF ratio (*p* = 0.91), MV SF ratio (*p* = 0.45), PEEP (*p* = 0.45), PIP (*p* = 0.87), MAP (*p* > 0.99), and vasopressor administration (*p* = 0.2) in animals with and without AKI were also not significantly associated with the development of AKI.

The SF ratio was calculable in 17 patients prior to MV (pre-MV SF ratio) and 44 patients after initiation of MV (MV SF ratio). Neither pre-MV SF ratio [AKI: *n* = 4, median 241.2, IQR (230.6–300.9) vs. Non-AKI: *n* = 13, median 242.5, (IQR 230.0–457.1)] (*p* = 0.91) nor post-MV SF ratio [AKI: *n* = 11, median 100.0, (IQR 98.5–112.5) vs. Non-AKI: *n* = 33, median 100.0, (IQR 96.0–108.8)] (*p* = 0.45) was significantly associated with the development of AKI.

In the subgroup of animals with normal baseline s[Cr], age (*p* = 0.39), sex (*p* = 0.24), CHF status (*p* = 0.11), duration of MV management (*p* = 0.36), length of hospitalization (*p* > 0.99), PEEP (*p* = 0.89), PIP (*p* = 0.19), MAP (*p* = 0.68), and vasopressor administration (*p* = 0.23) were not significantly associated with AKI development (Table 2).

In a univariable logistic regression model, baseline s[Cr] (OR 1.79, 95% CI 1.14–3.25, *p* = 0.01) and a diagnosis of CHF (OR 4.55, 95% CI 1.58–13.34, *p* = 0.0052) were significantly associated with AKI development. However, age (*p* = 0.089), indications of MV (*p* = 0.39), duration of MV management (*p* = 0.19), length of hospitalization (*p* = 0.12), pre-MV SpO_2_ (*p* = 0.78), MV SpO_2_ (*p* = 0.57), pre-MV SF ratio (*p* = 0.83), MV SF ratio (p = 0.83), PEEP (*p* = 0.55), PIP (*p* = 0.43), MAP (*p* = 0.87) were not significantly associated with the development of AKI in this cohort.

In multivariable logistic regression analysis including age, baseline s[Cr], and CHF status, duration of MV management, and length of hospitalization, none of the variables remained significantly associated with the development of AKI.

### AKI and clinical outcomes

**Figure 2:**
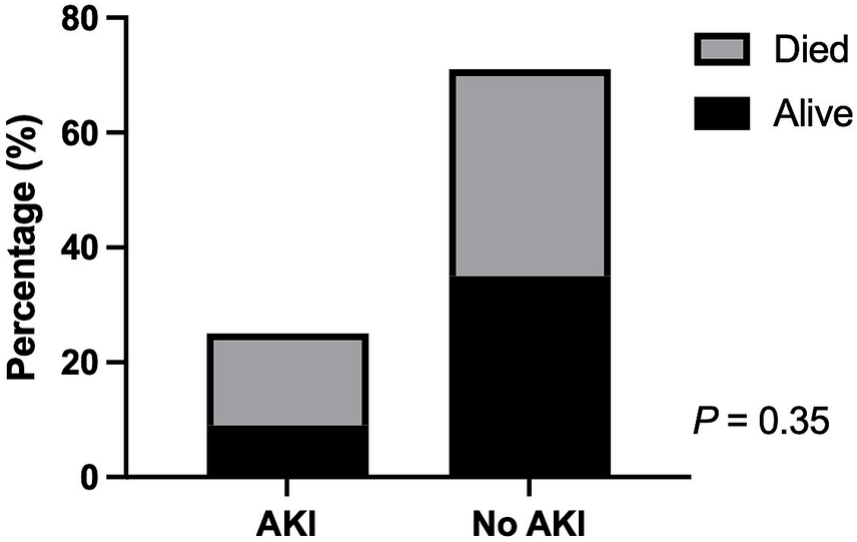
The proportions of survival and non-survival animals with or without acute kidney injury under mechanical ventilation.

The overall mortality in animals with AKI was 64% (16/25) and 50.7% (36/71) in animals without AKI (Figure 2). The MV weaning rate in animals with AKI was 40% (10/25) and 46.5% (33/71) without AKI. There was no significant difference in survival to discharge (*p* = 0.35) and successful weaning (*p* = 0.64) between animals with and without AKI (Table 3). The mortality rate of dogs with AKI was 66.7% (14/21) compared to 50% (30/60) of dogs without AKI (*p* = 0.21). The mortality rate in cats with AKI was 50% (2/4) and 54.6% (6/11) in cats without AKI (*p* > 0.99). In animals with CHF, AKI was diagnosed in 47.6% (10/21) of animals, and 50% (5/10) of animals with AKI and 45.5% (5/11) of animals without AKI died or were euthanized (*p* > 0.99). In animals without CHF, 20% (15/75) of animals developed AKI, and 73.3% (11/15) of animals with AKI died or were euthanized, and 51.7% (31/60) of animals without AKI died or were euthanized (*p* = 0.16).

**Table 3:** Baseline characteristics, AKI status, CHF status, respiratory parameters, and outcomes in animals (*n* = 96) managed by mechanical ventilation, categorized by survival status.

Baseline characteristics (all animals)		Survival		Non-survival	
Dogs	% (n/n)	45.7	(37/81)	54.3	(44/81)
Cats	% (n/n)	46.7	(7/15)	53.3	(8/15)
Overall	% (n/n)	45.8	(44/96)	54.2	(52/96)
Age	years	8	(4–10.8)	8	(4–10)
Sex male/female	n/n	19/25		29/23	
Baseline s[Cr]	mg/dL	0.6	(0.4–1.1)	0.75	(0.6–1.2)
AKI	% (n/n)	20.5	(9/44)	30.8	(16/52)
CHF	% (n/n)	25	(11/44)	19.2	(10/52)
Pre-MV SpO2	%	96^a^	(94–99)	93^a^	(89–97)
MV SpO2	%	100^b^	(97.8–100)	96^b^	(94–100)
Pre-MV SF ratio	-	250	(242.5–412.5)	237.5	(230–349.8)
MV SF ratio	-	100	(100–135.4)	98	(95–104.4)
PEEP	mmHg	5^c^	(3.5–6)	6^c^	(5–8.5)
PIP	mmHg	15	(13.1–18.5)	17	(15–21)
MAP	mmHg	83	(77–90)	84	(79–102)
Vasopressor use	% (n/n)	18.8^d^	(3/16)	58.1^d^	(18/31)
Duration of MV	hours	40	(24–66)	30	(15–50)
Length of hospitalization	days	5^e^	(4–7.8)	4^e^	(2–5)

Continuous variables were reported as median (interquartile range) or mean (standard deviation). Categorical variables were reported as percentage (*n*/total number).

AKI, acute kidney injury; s[Cr], serum creatinine concentration; Pre-MV SpO_2_, SpO_2_ measured prior to initiating mechanical ventilation management, MV SpO_2_, SpO_2_ measured within 24 h after the initiation of mechanical ventilation management; MV, mechanical ventilation; PEEP, positive end-expiratory pressure; PIP, peak inspiratory pressure; MAP, mean arterial pressure.

^a^*P* = 0.01; ^b^*P* = 0.0079; ^c^*P* = 0.047; ^d^*P* = 0.014; ^e^*P* < 0.0001.

The mortality rate of all animals with normal baseline s[Cr] was 53.8% (43/80), with death occurring in 61.1% (11/18) of those with AKI and 51.6% (32/62) of those without (*p* = 0.77, Table 4).

**Table 4:** Baseline characteristics, AKI status, CHF status, respiratory parameters, and outcomes in animals with normal baseline serum creatinine concentration (*n* = 80) managed by mechanical ventilation, categorized by survival status.

Baseline characteristics (normal baseline sCr)		Survival		Non-survival	
Dogs	% (n/n)	45.7	(32/70)	54.3	(38/70)
Cats	% (n/n)	50	(5/10)	50	(5/10)
Overall	% (n/n)	46.3	(37/80)	53.8	(43/80)
Age	Years	8	(4–10.5)	8	(4–11)
Sex male/female	n/n	14/23		23/20	
Baseline s[Cr]	mg/dL	0.6	(0.4–0.85)	0.7	(0.5–1)
AKI	% (n/n)	18.9	(7/37)	25.6	(11/43)
CHF	% (n/n)	18.9	(7/37)	9.3	(4/43)
Pre-MV SpO2%	%	97^a^	(+/− 2.3)	92.2^a^	(+/− 5.9)
MV SpO2	%	100^b^	(98–100)	96^b^	(94–100)
Pre-MV SF ratio	-	250	(250–466.7)	237.5	(230–242.5)
MV SF ratio	-	100^c^	(100–166.7)	97^c^	(94.5–104.4)
PEEP	mmHg	5^d^	(3.4–6)	6^d^	(5–9)
PIP	mmHg	15	(12–20.3)	17	(15–21)
MAP	mmHg	84	(77–99.8)	84	(79–110)
Vasopressor use	% (n/n)	8.3^e^	(1/12)	53.9^e^	(14/26)
Duration of MV	Hours	25	(16–64)	35	(15.5–64)
Length of hospitalization	Days	5^f^	(4–8)	4^f^	(2–5)

Continuous variables were reported as median (interquartile range) or mean (standard deviation). Categorical variables were reported as percentage (*n*/total number).

AKI, acute kidney injury; s[Cr], serum creatinine concentration; Pre-MV SpO_2_, SpO_2_ measured prior to initiating mechanical ventilation management, MV SpO_2_, SpO_2_ measured within 24 h after the initiation of mechanical ventilation management; MV, mechanical ventilation; PEEP, positive end-expiratory pressure; PIP, peak inspiratory pressure; MAP, mean arterial pressure.

^a^*P* = 0.0025; ^b^*P* = 0.0041; ^c^*P* = 0.031; ^d^*P* = 0.034; ^e^*P* = 0.012; ^f^*P* = 0.0012.

### Risk factors associated with the survival to discharge rate

**Figure 3:**
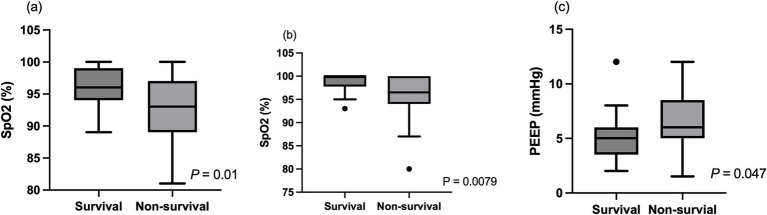
The association between peripheral oxygen saturation (SpO_2_), positive end-expiratory pressure (PEEP), and survival outcome in animals managed with mechanical ventilation. **(A)** Pre–mechanical ventilation SpO₂ was significantly higher in animals that survived to hospital discharge compared to non-survivors. **(B)** SpO₂ measured after initiation of MV was also significantly higher in survivors than in non-survivors. **(C)** PEEP was significantly lower in survivors than in non-survivors. In each plot, the box represents the interquartile range, the horizontal line within the box represents the median value, and the whiskers indicate the minimum and maximum values excluding outliers.

**Figure 4:**
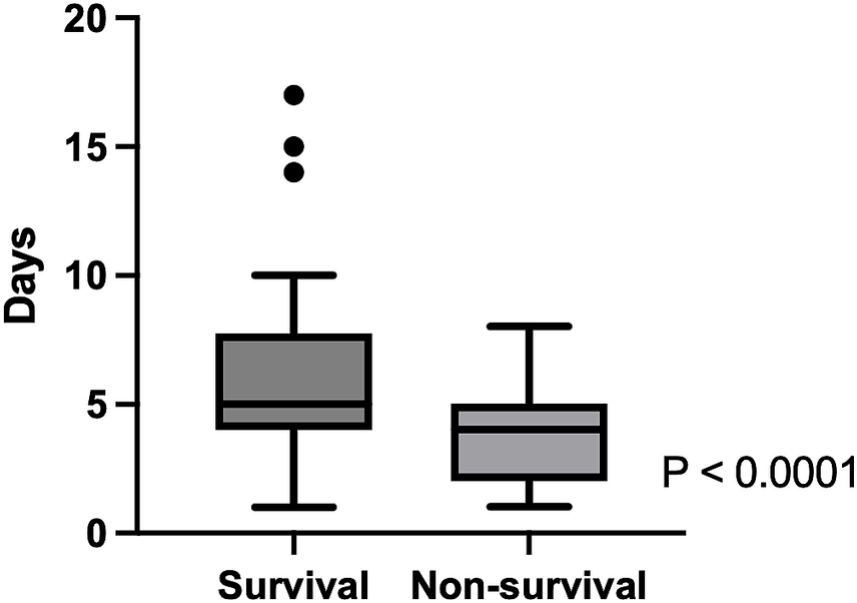
The association between length of hospitalization and survival outcome in dogs and cats managed with mechanical ventilation. Animals that survived to hospital discharge had a significantly longer duration of hospitalization than non-survivors (*p* < 0.0001). The box represents the interquartile range, the horizontal line within the box represents the median, and the whiskers indicate the minimum and maximum values excluding outliers.

The associations between various independent variables and the survival to discharge rate were investigated. Age (*p* = 0.91), sex (*p* = 0.31), AKI status (*p* = 0.35), indications of MV (p = 0.35), CHF status (*p* = 0.62), duration of MV (*p* = 0.74), baseline s[Cr] (*p* = 0.16), PIP (*p* = 0.33), MAP (*p* = 0.66), pre-MV SF ratio (*p* = 0.34), and MV SF ratio (*p* = 0.079) were not significantly associated with survival to discharge rate in animals (Table 3), while pre-MV SpO_2_ (*p* = 0.01), MV SpO_2_ (*p* = 0.0079), PEEP (*p* = 0.047), and vasopressor administration (*p* = 0.014) were significantly associated with the mortality rate (Figure 3). In addition, shorter hospitalization duration was significantly associated with higher mortality in these animals (*p* < 0.0001) (Figure 4).

Age (*p* = 0.66), sex (*p* = 0.18), baseline s[Cr] (*p* = 0.15), AKI status (*p* = 0.59), CHF status (*p* = 0.33), PEEP (*p* = 0.89), PIP (*p* = 0.19), MAP (*p* = 0.68), vasopressor administration (*p* = 0.23), and duration of MV (*p* = 0.91) were not significantly associated with survival to discharge in this cohort with the normal s[Cr] (Table 4), while pre-MV SpO_2_ (*p* = 0.0025), MV SpO_2_ (*p* = 0.0041) and length of hospitalization (*p* = 0.0012) were significantly associated with survival to discharge.

Baseline s[Cr] (*p* = 0.18), AKI status (*p* = 0.25), indications of MV (*p* = 0.43), pre-MV SF ratio (p = 0.68), and MV SF ratio (*p* = 0.8) were not significantly associated with the survival to discharge in univariable logistic regression analysis, whereas length of hospitalization (OR 0.61, 95% CI 0.45–0.78, *p* < 0.0001), pre-MV SpO_2_ (OR 0.82, 95% CI 0.67–0.96, *p* = 0.009), MV SpO_2_ (OR 0.77, 95% CI 0.6–0.94, *p* = 0.006) were. In the univariable logistic regression analysis, PEEP (*p* = 0.056), PIP (*p* = 0.27), and MAP (*p* = 0.92) were not significantly associated with survival to discharge.

A multivariable logistic regression analysis was also performed by including baseline s[Cr], duration of hospitalization, pre-MV SpO_2_, and PEEP. Of these variables included, pre-MV SpO₂ [odds ratio (OR) 0.79; 95% CI 0.61–0.96; *p* = 0.0135] and length of hospitalization (OR = 0.40; 95% CI, 0.19–0.66; *p* < 0.0001) were significantly associated with survival to discharge rate, whereas baseline s[Cr] and PEEP were not (*p* = 0.66).

## Discussion

In this retrospective study of mechanically ventilated animals, AKI was diagnosed in 26% of animals undergoing MV, with a similar incidence in dogs and cats. The overall mortality in animals with AKI was 64 and 50.7% in animals without AKI. The MV weaning rate in animals with AKI was 40 and 46.5% without AKI. There was no significant difference in survival to discharge or weaning success between animals with and without AKI. Of the animals with normal baseline s[Cr], 22.5% developed AKI, while 43.75% of animals with elevated baseline s[Cr] developed AKI with further elevation of s[Cr]. No significant associations between AKI development and the survival to discharge rate were identified in this subgroup of animals.

The incidence of AKI recorded in this study was comparable to that reported in human patients but higher than that previously reported in veterinary medicine (3, 8, 13, 20–22). Multiple mechanisms could explain the effects of MV with PPV on the development of AKI. These mechanisms include reduced renal perfusion secondary to elevated intrathoracic pressure with PPV, neurohormonal activation due to altered renal perfusion, and release of inflammatory mediators secondary to biotrauma of the respiratory system (7, 9–11). In the present study, MV settings and parameters were not recorded for many patients; therefore, the association between MV-related factors and AKI development could not be evaluated. Nevertheless, the lack of a significant association between the duration of MV and AKI suggests that MV-related factors may play a less significant role in AKI development than patient-related factors during MV management.

The presence of CHF was significantly associated with the development of AKI in univariable analyses. This variable, however, was not independently predictive of AKI after multivariable adjustment. This finding suggests that the association between CHF and AKI development may be confounded by various other factors including baseline s[Cr], age, and MV duration. In addition, transient azotemia in patients with CHF may reflect altered renal perfusion due to diuretic therapy rather than reduced renal function secondary to intrinsic kidney injury. In recent studies in dogs and cats with CHF, azotemia was not significantly associated with survival in these animals, probably due to decreased clearance of uremic toxins without significant loss of renal function due to intrinsic renal injury (26–28). This distinction is particularly important when interpreting the etiology of azotemia, as it may change the treatment strategies and potentially affect prognosis.

Baseline s[Cr] was significantly higher in animals that developed AKI in the univariable analysis but was not significantly associated with AKI in the multivariable analysis. This suggests that animals with pre-existing azotemia or kidney dysfunction may have a higher risk of developing AKI during MV. However, it was likely confounded by age and CHF status. Consequently, increases in s[Cr] during MV management could be due to the progression of pre-existing kidney dysfunction related to age or due to diuretic use rather than intrinsic kidney injury, though AKI with intrinsic kidney injury cannot be excluded. Given the retrospective nature of this study, it was not possible to distinguish between the progression of pre-existing kidney dysfunction or newly developed AKI attributable to MV management. This also highlights the limitations of renal functional markers, such as s[Cr], for diagnosing AKI. Using active kidney injury markers, such as urinary cystatin B or urinary neutrophil gelatinase-associated lipocalin, would help assess ongoing tubular injury and support the diagnosis of AKI (29–31).

Urine output during MV was not recorded in the majority of animals in this study. Since urine output is one of the key criteria for diagnosing AKI, it is plausible that the incidence of AKI was underestimated (32, 33). Conversely, elevations in s[Cr] may reflect inadequate renal perfusion or hydration, rather than intrinsic renal injury, potentially leading to an overestimation of AKI incidence due to intrinsic kidney injury. Nevertheless, the present study demonstrated a high incidence of increased s[Cr] following the initiation of MV management in dogs and cats, underscoring the importance of thorough assessment of renal function in these high-risk patients in clinical settings.

Contrary to our hypothesis and previous reports in human medicine, AKI was not associated with increased mortality in animals managed with MV (9, 21, 30, 34). This finding was persistent even in the subgroup of animals with CHF. Several potential factors may explain these negative findings. First, the overall survival to discharge rate of animals with MV management in this cohort was high, which may obscure the negative impact of AKI on survival. Second, the majority of AKI in this cohort was classified as IRIS grade I-II, which may indicate mild transient AKI with limited prognostic impact in such a high-mortality cohort. Third, early euthanasia due to severe primary diseases, especially with severe respiratory failure, may have precluded progression to more severe AKI or its negative consequences to the outcome. This interpretation is supported by the finding of the significant association between respiratory parameters (pre-MV and MV SpO_2_) and survival to discharge rate. In addition, shorter hospitalization duration was significantly associated with a higher mortality rate, likely reflecting early death or euthanasia in animals with severe respiratory failure. (35)

It is also important to note that a decent proportion of animals developed AKI despite having normal baseline s[Cr] prior to initiation of MV management. This finding is consistent with previously reported evidence that s[Cr] may remain within reference intervals until a significant decline in renal function has occurred, particularly in patients with reduced muscle mass, altered volume status, or acute hemodilution (19, 32, 33). This underscores the importance of serial renal monitoring and caution against reliance on a single serum creatinine measurement to assess the risk of progression or development of AKI in mechanically ventilated patients.

In the present study, s[Cr] was evaluated for up to 48 h after the initial measurement, and the progression and development of AKI beyond this timeframe could not be assessed. However, recent studies in human patients indicate that AKI associated with MV management most frequently occurs within the first 48 h after initiation of MV (13, 20). The primary pathophysiology of early (first 48 h) and late (48 h to 7 days) AKI appears to be different. Early AKI has been reported to be associated with impaired oxygenation, higher baseline s[Cr], and MV settings such as positive end-expiratory pressure, whereas late AKI is more likely related to the persistent hypoxemia and complications of the underlying diseases (20). As this present study focuses on early AKI, the observed association between higher baseline s[Cr] and AKI development is consistent with findings reported in human patients.

Several respiratory function parameters and MV settings were recorded in some animals included in this study. However, none of these parameters were significantly associated with AKI development. In human patients, several MV settings, including PEEP, PIP, respiratory system compliance, and driving pressure, have been reported to be associated with AKI development (7, 36). The lack of similar associations in the present study may be attributable to the relatively small sample size and inconsistent timing of data recording. Therefore, prospective studies including a larger population, standardized recording of MV settings, and detailed respiratory function parameters are warranted to determine the relationships between MV management and AKI development. Regarding the prediction of survival, pre-MV SpO_2_, MV SpO_2_, and PEEP were significantly associated with survival. Among these variables, pre-MV SpO_2_ was an independent predictor of survival, whereas PEEP was no longer significantly associated with outcome in the multivariable regression analysis. These findings indicate that the development of AKI was not predicted by lower SpO_2_, higher PEEP, or higher PIP. In contrast, some of these parameters were predictive of survival, suggesting that more severe respiratory failure, characterized by lower SpO_2_ and the need for more aggressive MV settings, was generally associated with a higher mortality rate.

Cardiovascular failure is another key contributor to AKI development in human patients (37). In the present study, MAP was not associated with AKI development or mortality. However, vasopressor administration was significantly associated with mortality. This suggests that animals requiring vasopressor or inotropes may have had more significant cardiovascular dysfunction despite preserved MAP, which may have contributed to the higher mortality rate. Nevertheless, vasopressor administration was not significantly associated with AKI development. This could be due to the improvement of renal perfusion with appropriate vasopressor use. In addition, the small sample size and retrospective nature of this study may have resulted in type II error. Further prospective analysis is warranted to determine the relationship between cardiovascular dysfunction, vasopressor use, and AKI development in mechanically ventilated animals.

There are some limitations in the present study. The retrospective design of this study limited the control of the type and timing of data acquisition. This limitation resulted in the exclusion of a large number of cases from data analyses. In addition, urine output measurement data were not available for many animals, potentially leading to underdiagnosis of AKI. Conversely, in the absence of concurrent urine output data, reversible AKI due to pre-renal causes cannot be differentiated from AKI due to intrinsic renal injury. Urinalysis and urine culture results were only available in a limited portion of our patients, which limited our ability to further assess the possibility of underlying urinary tract infection associated with AKI development. In addition, animals with pre-existing kidney disease were not excluded from this study due to the retrospective nature. This further justifies the need for future prospective studies to determine the incidence of AKI developing solely during MV. Some other important variables, including the types and volumes of fluid therapy, diuretic administration, sedation and anesthesia, hemodynamic parameters including plasma lactate concentration, and MV settings, were not included in this study, and all of these may influence renal perfusion and the risk of AKI development. The relatively small number of cats included in this study, compared with dogs, precluded findings of key differences between the species. Finally, euthanasia due to various reasons, such as a financially driven decision, may have confounded associations between AKI and survival to discharge rate.

Despite these limitations, the present study provided clinically relevant insight into AKI development in mechanically ventilated dogs and cats. AKI was common but not independently associated with survival in animals managed with MV, whereas SpO_2_, as a marker of respiratory failure severity, was significantly associated with survival. Prospective studies with urine output measurement and AKI active injury markers, along with detailed hemodynamic and respiratory parameters and ventilator data, are warranted to further elucidate the interactions between MV management and AKI development.

## Data Availability

The original contributions presented in the study are included in the article/Supplementary material, further inquiries can be directed to the corresponding author/s.
