# Supplementary material for: Incidence, risk factors, and prognosis of acute kidney injury in mechanically ventilated dogs and cats
Source: Front Vet Sci. 2026 May 1;13:1788257. doi: 10.3389/fvets.2026.1788257 (PMC13195400; doi:10.3389/fvets.2026.1788257)
Supplement: Supplementary file 1 [file Table_1.DOCX]

Supplemental table: Primary underlying diseases that were the main indications for mechanical ventilation in dogs and cats (n = 96). Data are presented as counts and percentages of the total study population. Individual patients could be diagnosed with more than one disease. As a result, the total number of reported diseases may exceed the total number of animals included in this study (n = 96).

| Primary | Count / Total  (n/n) | Percentage  (%) |
| --- | --- | --- |
| Congestive Heart Failure (CHF) | 21 / 96 | 21.9% |
| Pneumonia (Aspiration) | 14 / 96 | 14.6% |
| Pneumonia (Infectious/Broncho/Bacterial/blasto) | 10 / 96 | 10.4% |
| Unknown diagnosis | 9 / 96 | 9.4% |
| Cervical Myelopathy | 5 / 96 | 5.2% |
| Non-Cardiogenic Pulmonary Edema (NCPE) | 5 / 96 | 5.2% |
| Tracheal disease (Collapse/Tear) | 4 / 96 | 4.2% |
| Pulmonary Contusion | 4 / 96 | 4.2% |
| Brachycephalic Obstructive Airway Syndrome (BOAS) | 3 / 96 | 3.1% |
| Post-cardiopulmonary Arrest | 3 / 96 | 3.1% |
| Intracranial disease | 3 / 96 | 3.1% |
| Laryngeal paralysis | 2 / 96 | 2.1% |
| Lower Airway Disease (Unknown etiology) | 2 / 96 | 2.1% |
| Seizures | 2 / 96 | 2.1% |
| Smoke inhalation | 2 / 96 | 2.1% |
| AA-luxation | 1 / 96 | 1.04% |
| Blastomycosis | 1 / 96 | 1.04% |
| Heat Stroke | 1 / 96 | 1.04% |
| Ivermectin intoxication | 1 / 96 | 1.04% |
| NAC toxicity | 1 / 96 | 1.04% |
| Pneumothorax | 1 / 96 | 1.04% |
| Post-ACDO | 1 / 96 | 1.04% |
| Susp PTE | 1 / 96 | 1.04% |
| Susp diaphragmatic dysfunction | 1 / 96 | 1.04% |
| Susp rodenticide toxicity | 1 / 96 | 1.04% |
| Thoracic spinal fracture | 1 / 96 | 1.04% |
